# Factors Associated With Preoperative Radiological Tumor Size Underestimation in Clinical T1–2 Breast Cancer Patients

**DOI:** 10.1155/tbj/3726326

**Published:** 2026-06-26

**Authors:** Jungbin Kim, Yujin Lee, Sam-Youl Yoon, Hyunjin Cho, Ye Young Seo, Ji-Young Kim, Young-Joo Shin, Kyeongmee Park, Geumhee Gwak

**Affiliations:** ^1^ Department of Surgery, College of Medicine, Sanggye Paik Hospital, Inje University, Seoul, South Korea, inje.ac.kr; ^2^ Department of Nuclear Medicine, College of Medicine, Sanggye Paik Hospital, Inje University, Seoul, South Korea, medcol.mw; ^3^ Department of Radiology, College of Medicine, Sanggye Paik Hospital, Inje University, Seoul, South Korea, medcol.mw; ^4^ Department of Radiation Oncology, College of Medicine, Sanggye Paik Hospital, Inje University, Seoul, South Korea, medcol.mw; ^5^ Department of Pathology, College of Medicine, Sanggye Paik Hospital, Inje University, Seoul, South Korea, medcol.mw

**Keywords:** body mass index, breast neoplasms, carcinoma, histology, lobular, radiology

## Abstract

**Background:**

Accurate preoperative tumor size measurement is essential for determining optimal surgical margins in breast cancer patients. Thus, this study aimed to evaluate the factors associated with preoperative radiological tumor underestimation in clinical T (cT) Stage 1–2 breast cancer patients.

**Methods:**

We retrospectively reviewed the data of 365 cT1–2 breast cancer patients. Radiological tumor size was defined as the larger dimension on ultrasonography or magnetic resonance imaging. A pathological‐to‐radiological tumor size ratio > 1.2 or a tumor size discrepancy (pathology minus radiology) ≥ 5 mm was considered indicative of radiological underestimation. Preoperative variables, including age, body mass index (BMI), cT stage, maximum standardized uptake value of tumor, molecular subtype, and histologic subtype and grade, were analyzed to identify associated factors. Tumor size discrepancy (pathology minus radiology) was compared across subgroups, and the difference between pathological and radiological tumor size measurements was evaluated in each subgroup.

**Results:**

A BMI ≥ 25 kg/m^2^ (*p* = 0.006) and invasive lobular carcinoma (ILC) histology (*p* = 0.030) were associated with a pathological‐to‐radiological tumor size ratio > 1.2. A BMI ≥ 25 kg/m^2^ (*p* = 0.015), ILC histology (*p* = 0.022), and cT stage (*p* = 0.027) were associated with a tumor size discrepancy (pathology minus radiology) ≥ 5 mm. Significant differences in tumor size discrepancy between patients with a BMI ≥ 25 kg/m^2^ and those with a BMI < 25 kg/m^2^ (*p* = 0.003) and between patients with an ILC and those with non‐ILC (*p* = 0.041) were observed. Bland–Altman analysis showed radiological underestimation in ILCs (4.3 mm).

**Conclusion:**

In cT1–2 breast cancer patients, a BMI ≥ 25 kg/m^2^ and/or an ILC are predictive of radiological tumor underestimation, warranting supplemental imaging and intraoperative margin assessment.

## 1. Introduction

Accurate preoperative tumor‐size assessment is essential for optimal breast cancer management and serves as a key determinant for surgical planning in early‐stage disease [1]. Clinical T (cT) stage 1–2 breast cancers account for the majority of newly diagnosed cases, and breast‐conserving surgery (BCS) is usually the preferred treatment option when oncologically appropriate [2]. However, the success of BCS depends heavily on achieving negative surgical margins while preserving acceptable cosmetic outcomes, and the extent of tissue resection required to obtain adequate margins is directly influenced by preoperative tumor size measurement [3]. Therefore, precise preoperative tumor assessment is essential for determining appropriate surgical margins and optimizing both oncological safety and cosmetic results [4, 5].

Notably, despite advances in imaging technology, significant discordance between preoperative radiological measurements and final pathological tumor size persists, highlighting a critical gap in current practices [6, 7]. Moreover, studies have consistently demonstrated that conventional imaging modalities, such as mammography (MG) and ultrasonography (US), frequently underestimate actual tumor dimensions [8, 9]. This radiological underestimation carries significant clinical implications, as any underestimation directly correlates with higher positive margin rates and often necessitates reoperation after the initial BCS [10].

Meanwhile, the consequences of tumor size underestimation extend beyond immediate surgical outcomes. Radiological tumor size underestimation decreases the success rates for repeat surgeries, especially when the pathological tumor size exceeds the radiological measurements by over 50% [11]. Thus, these findings underscore the need to identify preoperative factors that contribute to significant tumor size discrepancies. Several previous studies have investigated factors associated with radiological tumor size underestimation, and invasive lobular carcinoma (ILC) histology has been consistently identified as a relevant factor [12, 13]. To further extend the scope of previous research, this study included additional variables. Specifically, body mass index (BMI), which has been associated with breast imaging accuracy [14, 15], and primary tumor (PT) maximum standardized uptake value (SUVmax) on preoperative ^18^F‐fluorodeoxyglucose positron emission tomography/computed tomography (FDG PET/CT), which reflects tumor metabolic activity and growth patterns [16, 17], were incorporated into the analysis.

Identifying preoperative predictors of significant radiological underestimation is crucial for improving both initial and long‐term surgical outcomes. Accordingly, this study aimed to investigate the preoperatively assessable factors associated with radiological tumor underestimation in cT1–2 breast cancer patients to enhance assessment accuracy and inform surgical decision‐making.

## 2. Materials and Methods

### 2.1. Patient Selection

This study retrospectively reviewed the medical records of 365 patients newly diagnosed with invasive breast cancer at our institution between January 2009 and June 2023. Patients with cT1–2 stage breast cancer were included based on the larger tumor size measured by preoperative US or magnetic resonance imaging (MRI). Patients who received neoadjuvant chemotherapy were excluded, as this treatment could alter the pathological tumor size.

During the study period, FDG PET/CT was performed in all patients diagnosed with breast cancer, regardless of the presence or absence of suspected systemic metastasis, either preoperatively or postoperatively. Patients without preoperative FDG PET/CT imaging were excluded, as PT SUVmax was used as a variable.

Patients were excluded if tumor size could not be reliably assessed on preoperative US or MRI or on postoperative pathological examination.

### 2.2. Variables

Clinical, histological, and imaging data were collected. Preoperatively assessable variables, including age, BMI, cT stage, PT SUVmax, histologic subtype, molecular subtype, and histologic grade (HG), were extracted from the medical records. Tumor size was measured using the preoperative US and/or MRI, with the larger dimension used to assign the cT stage when the two modalities differed. Staging followed the American Joint Committee on Cancer Staging Manual, 8th Edition, guidelines [18]. Tumors with > 90% lobular morphology and loss of E‐cadherin were classified as the lobular subtype. Tumors without a lobular phenotype or those with mixed lobular and nonlobular features were classified as the nonlobular subtype. Estrogen receptor (ER), progesterone receptor (PR), human epidermal growth factor 2 (HER2), and Ki‐67 expression levels were assessed by immunohistochemistry. Molecular subtypes were defined using immunohistochemical surrogate markers as follows: Luminal A: ER‐positive and/or PR‐positive, HER2‐negative, with a Ki‐67 index ≤ 20%; Luminal B: ER‐positive and/or PR‐positive with HER2‐negative and a Ki‐67 index > 20%, or ER‐positive and/or PR‐positive with HER2‐positive regardless of the Ki‐67 index; HER2‐positive: ER‐negative, PR‐negative, and HER2‐positive; and triple‐negative: ER‐negative, PR‐negative, and HER2‐negative. HG was determined using the Nottingham histologic scoring system. Pathology assessments were performed by a single pathologist with over 20 years of experience. All preoperative evaluations were performed within 1 month before the date of surgery.

### 2.3. Bilateral Breast and Axillary US

The bilateral breast and axillary US was performed by a single radiologist with over 20 years of experience, using a high‐frequency linear array transducer (5–12 or 4–18 MHz) on a Philips IU‐22 or EPIQ 7 US system (Philips Medical Systems, Bothell, WA, USA). Tumor dimensions were determined by measuring the maximum diameter of the lesion in multiple orthogonal planes (sagittal, transverse, and anteroposterior), and the largest dimension was recorded.

### 2.4. Dynamic Contrast‐Enhanced MRI

All MRI examinations were performed using either a Siemens MAGNETOM Avanto 1.5T or Skyra 3T scanner (Siemens Healthcare, Erlangen, Germany) equipped with a dedicated four‐channel bilateral breast coil. The protocol included the following sequences: axial turbo spin‐echo and fat‐suppressed T2‐weighted sequences (3 mm slice thickness), axial T1‐weighted sequences (3 mm slice thickness), axial diffusion‐weighted imaging with *b*‐values of 0 and 1000 s/mm^2^, and axial dynamic contrast‐enhanced T1‐weighted fat‐suppressed sequences consisting of one precontrast and five postcontrast dynamic series (1 mm slice thickness). Gadoterate meglumine (Dotarem; Guerbet LLC, Villepinte, France) was injected intravenously at a dose of 0.1 mmol/kg body weight through an antecubital vein, followed by a 20 mL saline flush. Postprocessing involved generating subtraction images between the precontrast, early postcontrast, and delayed postcontrast series, followed by reformatted axial and sagittal maximum‐intensity projections. All MRI examinations were reviewed by a single radiologist with over 20 years of experience. Tumor size was measured using an electronic digital caliper, and the largest dimension was recorded.

### 2.5. Statistical Analysis

The associations between variables and pathological‐to‐radiological tumor size ratio (≤ 1.2 vs. > 1.2), as well as tumor size discrepancy (pathology minus radiology, < 5 mm vs. ≥ 5 mm), were evaluated separately. Univariate analyses (chi‐square test and Fisher’s exact test), multivariate logistic regression analyses, and multiple linear regression analyses were performed to identify independent variables with tumor size discrepancy. The Mann–Whitney *U* test was used to compare tumor size discrepancy (pathology minus radiology) between two subgroups defined by independent factors. The degree of the relationship between the pathological and radiological tumor sizes was determined using Spearman’s rank correlation. Bland–Altman analysis was performed to assess the difference between pathological and radiological tumor size measurements in all patients and in each subgroup stratified by independent factors. A *p* value of < 0.05 was considered statistically significant. Data were analyzed using MedCalc Version 23.0.2 (MedCalc Software bvba, Ostend, Belgium).

## 3. Results

The clinical characteristics of all patients are presented in Table 1. Correlation between radiological and pathological tumor sizes and comparison of tumor sizes between US and MRI are shown in Table 2.

**TABLE 1:** Clinical characteristics of patients (*N* = 365).

**Characteristics**		***n* (%)**

Age (years)	≤ 40	19 (5.2%)
	41–50	118 (32.3%)
	51–60	101 (27.7%)
	61–70	73 (20.0%)
	≥ 71	54 (14.8%)

BMI (kg/m^2^)	< 23	137 (37.5%)
	≥ 23 and < 25	87 (23.8%)
	≥ 25 and < 30	105 (28.8%)
	≥ 30	36 (9.9%)

cT stage	1	208 (57.0%)
	2	157 (43.0%)

PT SUVmax	≤ 14.0	335 (91.8%)
	> 14.0	30 (8.2%)

Histologic subtype	IDC	315 (86.3%)
	ILC	21 (5.8%)
	Others	29 (7.9%)

Molecular subtype	Luminal A	126 (34.5%)
	Luminal B	163 (44.7%)
	HER2+	27 (7.4%)
	TN	49 (13.4%)

HG	1	35 (9.6%)
	2	153 (41.9%)
	3	177 (48.5%)

Tumor size discrepancy (pathology minus radiology, mm)	−29 to −10	31 (8.5%)
	−9 to −5	54 (14.8%)
	−4 to −1	116 (31.8%)
	0	50 (13.7%)
	1 to 4	64 (17.5%)
	5 to 9	24 (6.6%)
	10 to 45	26 (7.1%)

Abbreviations: BMI, body mass index; cT, clinical T; HER2+, human epidermal growth factor receptor 2‐positive breast cancer; HG, histologic grade; IDC, invasive ductal carcinoma; ILC, invasive lobular carcinoma; PT, primary tumor; SUVmax, maximum standardized uptake values; TN, triple‐negative breast cancer.

**TABLE 2:** Correlation between radiological and pathological tumor sizes and comparison of tumor sizes between US and MRI.

Modality	*N*	Correlation coefficient (*r*)
US	365	0.735^1^

MRI	269	0.710^1^

Comparison of tumor sizes	US > MRI	72
	US = MRI	91
	US < MRI	106

Abbreviations: MRI, magnetic resonance imaging; US, ultrasonography.

^1^Pearson’s correlation coefficient.

A BMI ≥ 25 kg/m^2^ (*p* = 0.006) and ILC histology (*p* = 0.030) were found to be significantly associated with a pathological‐to‐radiological tumor size ratio > 1.2 in both univariate and multivariate analyses (Table 3). A BMI ≥ 25 kg/m^2^ (*p* = 0.015), ILC histology (*p* = 0.022), and cT2 (*p* = 0.027) were found to be significantly associated with a tumor size discrepancy (pathology minus radiology) ≥ 5 mm in both univariate and multivariate analyses (Table 4).

**TABLE 3:** Univariate and multivariate analyses of factors associated with pathological tumor size exceeding radiological size by > 20% in cT1–2 breast cancer patients (*N* = 365).

Factors	*n*	Pathological‐to‐radiological tumor size ratio		*p* value	
		≤ 1.2 (*n* = 309)	> 1.2 (*n* = 56)	Univariate	Multivariate
Mean tumor size discrepancy (pathology minus radiology, mm)		−3.2	11.2		
Age (years)				0.510^1^	
≤ 40	19	15	4		
> 41	346	294	52		
BMI (kg/m^2^)				0.005^2^	0.006^4^
< 25	224	199	25		
≥ 25	141	110	31		
cT stage				0.394^2^	
1	208	179	29		
2	157	130	27		
PT SUVmax				0.073^2^	
≤ 14.0	335	287	48		
> 14.0	30	22	8		
Histologic subtype				0.019^2^	0.030^4^
Non‐ILC	344	295	49		
ILC	21	14	7		
Molecular subtype				0.333^3^	
TN	49	38	11		
Luminal B or HER2+	190	163	27		
Luminal A	126	108	18		
HG				0.027^1^	0.084^4^
1	35	34	1		
2–3	330	275	55		

Abbreviations: BMI, body mass index; cT, clinical T; HER2+, human epidermal growth factor receptor 2‐positive breast cancer; HG, histologic grade; ILC, invasive lobular carcinoma; PT, primary tumor; SUVmax, maximum standardized uptake values; TN, triple‐negative breast cancer.

^1^Fisher’s exact test.

^2^Chi‐square test.

^3^Chi‐square test for trend.

^4^Multivariate logistic regression analysis.

**TABLE 4:** Univariate and multivariate analyses of factors associated with pathological tumor size exceeding radiological size by ≥ 5 mm in cT1–2 breast cancer patients (*N* = 365).

Factors	*n*	Tumor size discrepancy (pathology minus radiology)		*p* value	
		< 5 mm (*n* = 315)	≥ 5 mm (*n* = 50)	Univariate	Multivariate
Mean tumor size discrepancy (pathology minus radiology, mm)		−3.1	12.4		
Age (years)				0.310^1^	
≤ 40	19	15	4		
> 41	346	300	46		
BMI (kg/m^2^)				0.016^2^	0.015^4^
< 25	224	201	23		
≥ 25	141	114	27		
cT stage				0.001^2^	0.027^4^
1	208	190	18		
2	157	125	32		
PT SUVmax				0.031^2^	0.199^4^
≤ 14.0	335	293	42		
> 14.0	30	22	8		
Histologic subtype				0.041^2^	0.022^4^
Non‐ILC	344	300	44		
ILC	21	15	6		
Molecular subtype				0.049^3^	0.249^4^
TN	49	39	10		
Luminal B or HER2+	190	162	28		
Luminal A	126	114	12		
HG				0.008^1^	0.998^4^
1	35	35	0		
2–3	330	280	50		

Abbreviations: BMI, body mass index; cT, clinical T; HER2+, human epidermal growth factor receptor 2‐positive breast cancer; HG, histologic grade; ILC, invasive lobular carcinoma; PT, primary tumor; SUVmax, maximum standardized uptake values; TN, triple‐negative breast cancer.

^1^Fisher’s exact test.

^2^Chi‐square test.

^3^Chi‐square test for trend.

^4^Multivariate logistic regression analysis.

Multiple linear regression analysis demonstrated that higher BMI, ILC histology, smaller radiological tumor size, and higher PT SUVmax were independently associated with greater radiological tumor size underestimation (Table 5). BMI (*β* = 0.297, *p* = 0.004), ILC histology (*β* = 5.948, *p* = 0.001), radiological tumor size (*β* = −0.177, *p* = 0.001), and PT SUVmax (*β* = 0.281, *p* = 0.003) remained significant.

**TABLE 5:** Multiple linear regression analysis of factors associated with tumor size discrepancy in cT1–2 breast cancer patients (*N* = 365).

Factors	Coefficient (*β*)	95% confidence interval	*p* value
BMI (kg/m^2^)	0.297	0.096 to 0.499	0.004
Histologic subtype (Non‐ILC vs. ILC)	5.948	2.445 to 9.451	0.001
Radiological tumor size (mm)	−0.177	−0.272 to −0.082	0.001
PT SUVmax	0.281	0.097 to 0.465	0.003
Molecular subtype (nonluminal A vs. luminal A)	−0.427	−2.345 to 1.491	0.662
HG (1 vs. 2–3)	2.061	−0.918 to 5.039	0.175

*Note:* *R* ^2^ = 0.098, Adjusted *R* ^2^ = 0.085.

Abbreviations: BMI, body mass index; cT, clinical T; HG, histologic grade; ILC, invasive lobular carcinoma; PT, primary tumor; SUVmax, maximum standardized uptake values.

The Mann–Whitney *U* test demonstrated significant differences in tumor size discrepancy (pathology minus radiology) between patients with a BMI ≥ 25 kg/m^2^ and those with a BMI < 25 kg/m^2^ (*p* = 0.003) and between patients with an ILC and those with non‐ILC (*p* = 0.041; Table 6).

**TABLE 6:** Comparison of tumor size discrepancy among cT1–2 breast cancer patients (*N* = 365).

			**Tumor size discrepancy (pathology minus radiology, mm)** **Mean, median (range)**	***p* value**

BMI (kg/m^2^)	< 25	(*n* = 224)	−1.6, −2 (−29–40)	0.003^1^
	≥ 25	(*n* = 141)	0.1, −1 (−28–45)	

Histologic subtype	Non‐ILC	(*n* = 344)	−1.3, −1 (−29–45)	0.041^1^
	ILC	(*n* = 21)	4.4, 0 (−10–37)	

Abbreviations: BMI, body mass index; cT, clinical T; ILC, invasive lobular carcinoma.

^1^Mann–Whitney *U* test.

**FIGURE 1:**
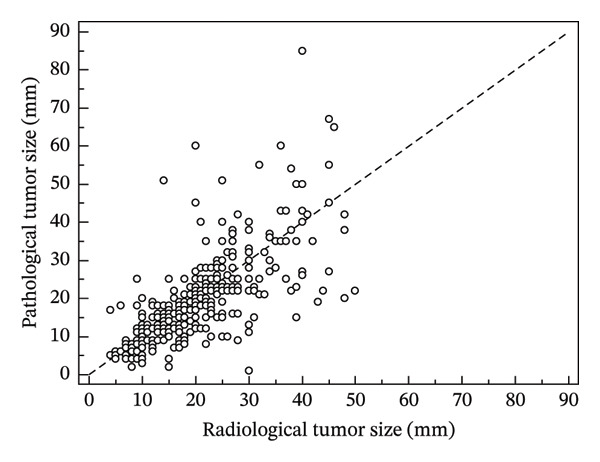
Scatter diagram of Spearman’s rank correlation showing the relationship between pathological and radiological tumor size (rho = 0.789, 95% confidence interval: 0.747 to 0.825; *p* < 0.001) (*N* = 365).

A strong overall correlation between the pathological and radiological tumor sizes was observed, as determined by Spearman’s rank correlation coefficient (Spearman’s rho = 0.789, *p* < 0.001; Figure 1).

**FIGURE 2:**
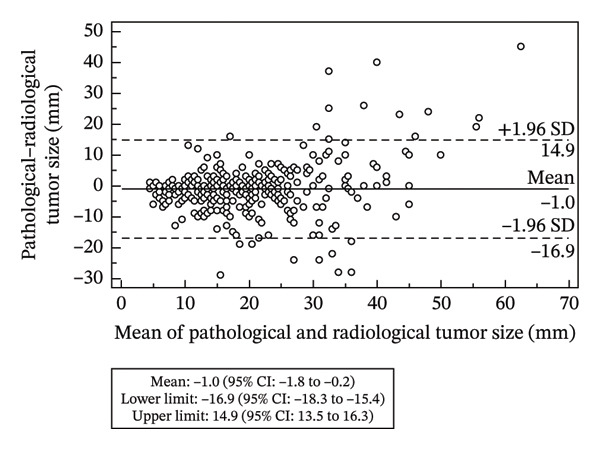
Bland–Altman plots for all patients (*N* = 365). Abbreviations: CI, confidence interval; SD, standard deviation.

**FIGURE 3:**
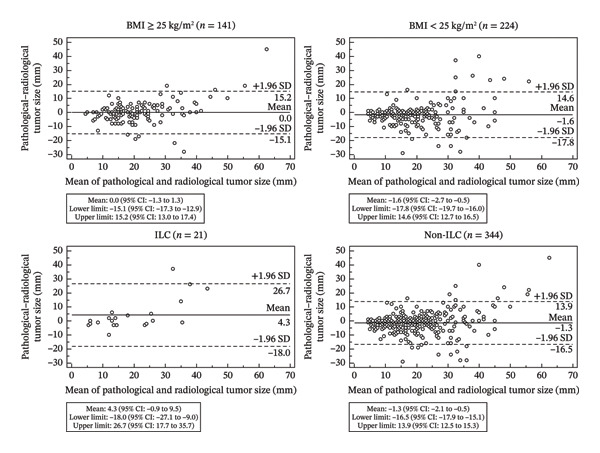
Bland–Altman plots for subgroups stratified by BMI or histologic subtype. Abbreviations: BMI, body mass index; CI, confidence interval; ILC, invasive lobular carcinoma; SD, standard deviation.

The Bland–Altman analysis for all patients showed a mean difference of −1.0 mm and limits of agreement (LoAs) ranging from −16.9 mm to 14.9 mm between the radiological and pathological measurements (Figure 2). In the subgroup analyses, patients with an ILC demonstrated radiological tumor underestimation (4.3 mm). In contrast, patients with a BMI ≥ 25 kg/m^2^ showed no systematic bias (0.0 mm). Meanwhile, radiological tumor overestimation was observed among patients with a BMI < 25 kg/m^2^ (−1.6 mm) and those with non‐ILC (−1.3 mm) (Figure 3). Figure 3 also presents the LoAs for each subgroup: BMI < 25 kg/m^2^, −17.8 to 14.6 mm; BMI ≥ 25 kg/m^2^, −15.1–15.2 mm; non‐ILC: −16.5 to 13.9 mm; and ILC: −18.0–26.7 mm.

## 4. Discussion

Accurate preoperative measurement of tumor size is crucial for optimal BCS, as a radiological underestimation correlates with positive margins and higher rates of reoperation [10, 19]. Therefore, by recognizing predictors of underestimation, surgeons can incorporate supplemental imaging techniques or intraoperative margin assessment to mitigate the risk of incomplete excision. In multiple analyses performed in this study, BMI and histologic subtype were consistently identified as significant predictors of radiological tumor underestimation, providing new insights into patient‐specific risk factors.

In this study, the Mann–Whitney *U* test showed no tendency for radiological tumor underestimation in the BMI ≥ 25 kg/m^2^ or ILC patient groups, whereas the Bland–Altman statistics demonstrated a tendency for radiological tumor underestimation in the ILC patient group. This discrepancy between the two statistical methods may be attributed to the different analytical approaches: the Mann–Whitney *U* test compares median values, whereas the Bland–Altman analysis assesses agreement between measurements using mean values and LoAs. Despite this inconsistency, both analyses consistently showed significantly smaller radiological tumor size measurements among patients with a BMI ≥ 25 kg/m^2^ and an ILC compared to those with a BMI < 25 kg/m^2^ and non‐ILC, respectively. Moreover, the univariate and multivariate analyses identified BMI ≥ 25 kg/m^2^ and ILC as independent predictors of significant tumor size underestimation (> 20% or ≥ 5 mm) on preoperative imaging relative to pathological size. Collectively, these findings indicate that a high BMI and ILC may be associated with radiological tumor underestimation.

Conventional imaging modalities, including MG and US, have been shown to underestimate tumor size [8, 9]. Gruber et al. reported that the tumor size could be significantly underestimated by US with a mean difference of 8 mm compared to pathological measurements [20]. Furthermore, Simpson et al. found similar degrees of tumor size underestimation with both MG and US [11]. Conversely, some studies have reported that tumor size tends to be overestimated by MRI measurements compared to pathological measurements [9, 21]. However, breast MRI is widely used preoperatively for breast cancer patients, making MRI‐derived measurements critically important. Therefore, we included MRI as a measurement modality for assessing tumor size. Nonetheless, despite the potential for MRI‐related overestimations, the MRI‐derived values were retained rather than excluded, allowing a comprehensive assessment of imaging errors and enhancing the explanatory power of our findings.

Many studies have investigated the association between obesity and breast cancer characteristics [22, 23]. However, research directly linking obesity to radiological–pathological tumor size discrepancy remains scarce. Nevertheless, the impact of obesity on imaging accuracy can be inferred from studies demonstrating degraded radiological performance in breast cancer screening as obesity levels increase [23, 24]. In addition, mammographic compression is less uniform in obese breasts, compromising two‐dimensional size measurements [24].

Several mechanisms may explain the relationship between higher BMI values and radiological underestimation. First, obesity increases the secretion of inflammatory cytokines, including insulin, IGF‐1, TNF‐α, and IL‐6, from adipocytes, which enhance tumor invasiveness and growth. These factors promote diffuse infiltration, making tumor boundaries less distinct on imaging and leading to underestimation [25, 26]. Moreover, obesity induces chronic inflammation in the breast, increasing adipocyte necrosis and macrophage‐driven crown‐like structure (CLS) formation in breast adipose tissue, and CLS‐mediated NF‐κB activation induce the upregulation of aromatase expression and local estrogen production [27]. Chronic inflammation‐induced estrogen promotes the proliferation and infiltration of breast cancer cells, resulting in microscopic invasive areas that are not clearly visible on imaging assessments but are measured as larger on pathology [28, 29]. In ER‐negative tumors, locally produced estrogen via obesity‐driven aromatase induction can also act through nonclassical mechanisms, such as G protein‐coupled ER 30 signaling and ER‐α36 variant‐mediated pathways [30, 31]. Moreover, cancer cells in obese patients tend to infiltrate adipose tissue irregularly, resulting in indistinct or partially missed margins on imaging [32, 33]. However, pathology can capture the full extent of infiltration, increasing the size discrepancy. Higher BMI values also contribute to greater compressed breast thickness during US, which limits probe penetration, reduces image resolution, and blurs tumor margins, leading to underestimation [34].

Multiple studies have demonstrated that ILCs tend to be underestimated in size by imaging modalities, such as US and MRI, among various breast cancer histologic subtypes. Pritt et al. reported that the pathological size was consistently underestimated across all histologic subtypes by US, with ILCs showing the greatest discrepancy [12]. According to Hovis et al., even MRI, which generally overestimates tumor size in breast cancer patients [9], tends to underestimate the size of ILCs, although to a lesser degree than that of US [13]. ILC often presents as a single‐file, diffuse infiltration of tumor cells without forming a distinct mass, making delineation of lesion boundaries difficult on US and MRI, leading to underestimation of the true extent [35]. Furthermore, compared to invasive ductal carcinoma, ILC induces a minimal desmoplastic stromal response, resulting in reduced collagen deposition and a weaker contrast between tumor and normal tissue on imaging, particularly via US [36].

In this study, univariate and multivariate analyses of factors associated with pathological tumor size exceeding radiological size by ≥ 5 mm identified higher cT stage as a significant factor associated with radiological tumor underestimation. This finding is inconsistent with those previous studies [6, 7] and may be attributable to the increasing absolute magnitude of tumor size discrepancy with advancing cT stage. Therefore, the association between higher cT stage and radiological tumor underestimation observed in the univariate and multivariate analyses may have limited clinical significance.

Meanwhile, although radiological tumor size and PT SUVmax were not significantly associated with radiological tumor underestimation in the univariate and multivariate analyses, multiple linear regression analysis demonstrated that smaller radiological tumor size and higher PT SUVmax were associated with a greater tendency toward radiological tumor size underestimation. These findings are consistent with those of previous studies [6, 7, 16, 17] and support the clinical relevance of radiological tumor size and PT SUVmax in relation to tumor size underestimation, thereby providing a rationale for further related investigations.

Previous studies [37, 38] have suggested a tendency toward radiological underestimation of tumor size in the luminal A subtype; however, this was not statistically significant in our study. This discrepancy may be explained by differences in tumor biology and analytical approaches. In particular, the use of a threshold‐based definition of size discrepancy (> 20% or ≥ 5 mm) may have limited the detection of subtle differences between molecular subtypes. In addition, as our cohort was restricted to early‐stage (cT1–2) breast cancer, the relatively limited extent of tumor infiltration may have reduced the impact of subtype‐specific growth patterns on size underestimation.

Based on our findings, both US and MRI significantly underestimated tumor size in patients with a BMI ≥ 25 kg/m^2^ or ILC, suggesting that supplementary imaging techniques or biopsy‐based assessments are required for the precise evaluation of tumor extent. The use of preoperative cone‐beam breast computed tomography [39], intraoperative US [40], or emerging modalities such as optical coherence tomography [41] and radiofrequency spectroscopy [42] for real‐time margin evaluation can help reduce residual disease rates. Moreover, simultaneously planning for wider excision margins—associated with a lower risk of residual disease—along with intraoperative frozen‐section analysis of the resection margins in these high‐risk patients can further reduce re‐excision rates [43, 44].

Despite the valuable insights from this study, the retrospective design and single‐center setting may limit the generalizability of these findings to broader clinical populations, particularly given potential selection bias arising from limited demographic diversity. Therefore, a prospective multicenter study is warranted to improve the validity of these findings. The relatively small number of patients with ILC limits the strength of the analysis and warrants cautious interpretation of the observed tendency for underestimation of tumor size on preoperative imaging in ILC compared with non‐ILC, highlighting the need for studies with larger patient cohorts to validate these findings. As this study included patients over a long period (2009–2023), some imaging studies were performed using older‐generation scanners, which may be associated with lower image quality compared with current technologies. This heterogeneity across the study period could have influenced the accuracy of tumor size estimation and should be considered when interpreting our findings.

## 5. Conclusion

In conclusion, cT1–2 breast cancer patients with a BMI ≥ 25 kg/m^2^ and/or an ILC are more likely to have pathological tumor sizes over 20% larger than those estimated using imaging modalities. Therefore, supplemental imaging and intraoperative margin assessment are required to optimize surgical planning and reduce re‐excision rates.

## Author Contributions

Jungbin Kim conceived the study and drafted the initial manuscript. Hyunjin Cho designed the study methodology. Ye Young Seo, Ji‐Young Kim, Young‐Joo Shin, and Kyeongmee Park performed the data collection, while Yujin Lee and Sam‐Youl Yoon conducted the data analysis. Geumhee Gwak supervised the study.

## Funding

No funding was received for this research.

## Disclosure

All authors reviewed and provided feedback on the first draft and approved the final manuscript.

## Ethics Statement

The Institutional Review Board of Inje University Sanggye Paik Hospital approved this study (approval no. 2024‐10‐007).

## Consent

The Institutional Review Board of Inje University Sanggye Paik Hospital waived the requirement for informed consent due to the retrospective design of the study.

## Conflicts of Interest

The authors declare no conflicts of interest.

## Data Availability

The datasets generated and analyzed during the current study are not publicly available due to ethical restrictions but are available from the corresponding author upon reasonable request.

## References

[1] Pop C. F. , Stanciu-Pop C. , Drisis S. et al., The Impact of Breast MRI Workup on Tumor Size Assessment and Surgical Planning in Patients With Early Breast Cancer, Breast Journal. (2018) 24, no. 6, 927–933, 10.1111/tbj.13104. 30076661

[2] van Maaren M. C. , Strobbe L. J. A. , Koppert L. B. , Poortmans P. M. P. , and Siesling S. , Nationwide Population-Based Study of Trends and Regional Variation in Breast-Conserving Treatment for Breast Cancer, British Journal of Surgery. (2018) 105, no. 13, 1768–1777, 10.1002/bjs.10951. 30091459

[3] Pleijhuis R. G. , Graafland M. , de Vries J. , Bart J. , de Jong J. S. , and van Dam G. M. , Obtaining Adequate Surgical Margins in breast-conserving Therapy for Patients With Early-Stage Breast Cancer: Current Modalities and Future Directions, Annals of Surgical Oncology. (2009) 16, no. 10, 2717–2730, 10.1245/s10434-009-0609-z. 19609829 PMC2749177

[4] Moran M. S. , Schnitt S. J. , Giuliano A. E. et al., Society of Surgical Oncology-American Society for Radiation Oncology Consensus Guideline on Margins for Breast-Conserving Surgery With Whole-Breast Irradiation in Stages I and II Invasive Breast Cancer, International Journal of Radiation Oncology Biology Physics. (2014) 88, no. 3, 553–564, 10.1016/j.ijrobp.2013.11.012. 24521674 PMC4790083

[5] Gozali A. and Piper M. , Optimizing Outcomes in Oncoplastic Breast-Conserving Surgery, Journal of Clinical Medicine. (2025) 14, no. 13, 10.3390/jcm14134806. PMC12250907 40649180

[6] Hamza A. , Khawar S. , Sakhi R. et al., Factors Affecting the Concordance of Radiologic and Pathologic Tumor Size in Breast Carcinoma, Ultrasound. (2019) 27, no. 1, 45–54, 10.1177/1742271x18804278. 30774698 PMC6362536

[7] Liu Y. , Liao X. , He Y. et al., Tumor Size and Stage Assessment Accuracy of MRI and Ultrasound Versus Pathological Measurements in Early Breast Cancer Patients, BMC Women′s Health. (2025) 25, no. 1, 10.1186/s12905-025-03679-2. PMC11969697 40186264

[8] Daly A. E. , Anderman K. J. , Holt L. R. et al., Sizing It Up: Concordance Between Breast Imaging and Pathologically Determined Tumor Measurement, Annals of Surgical Oncology. (2025) 32, no. 12, 8686–8692, 10.1245/s10434-025-17663-5. 40522576

[9] Azhdeh S. , Kaviani A. , Sadighi N. , and Rahmani M. , Accurate Estimation of Breast Tumor Size: A Comparison Between Ultrasonography, Mammography, Magnetic Resonance Imaging, and Associated Contributing Factors, Eur J Breast Health. (2021) 17, no. 1, 53–61, 10.4274/ejbh.2020.5888. 33796831 PMC8006785

[10] Schultek G. , Gerber B. , Reimer T. et al., Radiological Underestimation of Tumor Size as a Relevant Risk Factor for Positive Margin Rate in Breast-Conserving Therapy of Pure Ductal Carcinoma in Situ (DCIS), Cancers (Basel). (2022) 14, no. 10, 10.3390/cancers14102367. PMC9139437 35625972

[11] Simpson D. , Allan J. , and McFall B. , Radiological Underestimation of Tumor Size Influences the Success Rate of Re-Excision After Breast-Conserving Surgery, European Journal of Breast Health. (2021) 17, no. 4, 363–370, 10.4274/ejbh.galenos.2021.2021-4-7. 34651116 PMC8496123

[12] Pritt B. , Ashikaga T. , Oppenheimer R. G. , and Weaver D. L. , Influence of Breast Cancer Histology on the Relationship Between Ultrasound and Pathology Tumor Size Measurements, Modern Pathology. (2004) 17, no. 8, 905–910, 10.1038/modpathol.3800138. 15105809

[13] Hovis K. K. , Lee J. M. , Hippe D. S. et al., Accuracy of Preoperative Breast MRI Versus Conventional Imaging in Measuring Pathologic Extent of Invasive Lobular Carcinoma, Journal of Breast Imaging. (2021) 3, no. 3, 288–298, 10.1093/jbi/wbab015. 34061121 PMC8139612

[14] Elmore J. G. , Carney P. A. , Abraham L. A. et al., The Association Between Obesity and Screening Mammography Accuracy, Archives of Internal Medicine. (2004) 164, no. 10, 1140–1147, 10.1001/archinte.164.10.1140. 15159273 PMC3143016

[15] Wang Y. , Li Y. , Song Y. et al., Comparison of Ultrasound and Mammography for Early Diagnosis of Breast Cancer Among Chinese Women with Suspected Breast Lesions: A Prospective Trial, Thoracic Cancer. (2022) 13, no. 22, 3145–3151, 10.1111/1759-7714.14666. 36177910 PMC9663682

[16] Jung N. Y. , Kim S. H. , Choi B. B. , Kim S. H. , and Sung M. S. , Associations Between the Standardized Uptake Value of (18)F-FDG PET/CT and the Prognostic Factors of Invasive Lobular Carcinoma: in Comparison With Invasive Ductal Carcinoma, World Journal of Surgical Oncology. (2015) 13, no. 1, 10.1186/s12957-015-0522-9. PMC4371618 25889560

[17] Shigematsu H. , Kadoya T. , Masumoto N. et al., Role of FDG-PET/CT in Prediction of Underestimation of Invasive Breast Cancer in Cases of Ductal Carcinoma in Situ Diagnosed at Needle Biopsy, Clinical Breast Cancer. (2014) 14, no. 5, 358–364, 10.1016/j.clbc.2014.04.006. 24962555

[18] Teichgraeber D. C. , Guirguis M. S. , and Whitman G. J. , Breast Cancer Staging: Updates in the AJCC Cancer Staging Manual, 8th Edition, and Current Challenges for Radiologists, From the AJR Special Series on Cancer Staging, American Journal of Roentgenology. (2021) 217, no. 2, 278–290, 10.2214/ajr.20.25223. 33594908

[19] Meier-Meitinger M. , Rauh C. , Adamietz B. et al., Accuracy of Radiological Tumour Size Assessment and the Risk for Re-Excision in a Cohort of Primary Breast Cancer Patients, European Journal of Surgical Oncology. (2012) 38, no. 1, 44–51, 10.1016/j.ejso.2011.10.008. 22032911

[20] Gruber I. V. , Rueckert M. , Kagan K. O. et al., Measurement of Tumour Size With Mammography, Sonography and Magnetic Resonance Imaging as Compared to Histological Tumour Size in Primary Breast Cancer, BMC Cancer. (2013) 13, no. 1, 10.1186/1471-2407-13-328. PMC3704854 23826951

[21] Leddy R. , Irshad A. , Metcalfe A. et al., Comparative Accuracy of Preoperative Tumor Size Assessment on Mammography, Sonography, and MRI: Is the Accuracy Affected by Breast Density or Cancer Subtype?, Journal of Clinical Ultrasound. (2016) 44, no. 1, 17–25, 10.1002/jcu.22290. 26294391

[22] Blair C. K. , Wiggins C. L. , Nibbe A. M. et al., Obesity and Survival Among a Cohort of Breast Cancer Patients Is Partially Mediated by Tumor Characteristics, NPJ Breast Cancer. (2019) 5, no. 1, 10.1038/s41523-019-0128-4. PMC6775111 31602394

[23] Toro A. L. , Costantino N. S. , Shriver C. D. , Ellsworth D. L. , and Ellsworth R. E. , Effect of Obesity on Molecular Characteristics of Invasive Breast Tumors: Gene Expression Analysis in a Large Cohort of Female Patients, BMC Obes. (2016) 3, no. 1, 10.1186/s40608-016-0103-7. PMC4850667 27148454

[24] Guest A. R. , Helvie M. A. , Chan H. P. , Hadjiiski L. M. , Bailey J. E. , and Roubidoux M. A. , Adverse Effects of Increased Body Weight on Quantitative Measures of Mammographic Image Quality, American Journal of Roentgenology. (2000) 175, no. 3, 805–810, 10.2214/ajr.175.3.1750805. 10954471

[25] Hillers L. E. , D′Amato J. V. , Chamberlin T. , Paderta G. , and Arendt L. M. , Obesity-Activated Adipose-Derived Stromal Cells Promote Breast Cancer Growth and Invasion, Neoplasia. (2018) 20, no. 11, 1161–1174, 10.1016/j.neo.2018.09.004. 30317122 PMC6187054

[26] Ibrahim A. S. , El-Shinawi M. , Sabet S. , Ibrahim S. A. , and Mohamed M. M. , Role of Adipose Tissue-Derived Cytokines in the Progression of Inflammatory Breast Cancer in Patients With Obesity, Lipids in Health and Disease. (2022) 21, no. 1, 10.1186/s12944-022-01678-y. PMC9351154 35927653

[27] Morris P. G. , Hudis C. A. , Giri D. et al., Inflammation and Increased Aromatase Expression Occur in the Breast Tissue of Obese Women With Breast Cancer, Cancer Prevention Research. (2011) 4, no. 7, 1021–1029, 10.1158/1940-6207.Capr-11-0110. 21622727 PMC3131426

[28] Vazquez R. G. , Abrahamsson A. , Jensen L. D. , and Dabrosin C. , Estradiol Promotes Breast Cancer Cell Migration via Recruitment and Activation of Neutrophils, Cancer Immunology Research. (2017) 5, no. 3, 234–247, 10.1158/2326-6066.Cir-16-0150. 28159748

[29] Zhu M. , Pi Y. , Jiang Z. et al., Application of Deep Learning to Identify Ductal Carcinoma in Situ and Microinvasion of the Breast Using Ultrasound Imaging, Quantitative Imaging in Medicine and Surgery. (2022) 12, no. 9, 4633–4646, 10.21037/qims-22-46. 36060588 PMC9403599

[30] Ariazi E. A. , Brailoiu E. , Yerrum S. et al., The G protein-Coupled Receptor GPR30 Inhibits Proliferation of Estrogen Receptor-Positive Breast Cancer Cells, Cancer Research. (2010) 70, no. 3, 1184–1194, 10.1158/0008-5472.Can-09-3068. 20086172 PMC2879282

[31] Zhang J. , Li G. , Li Z. et al., Estrogen-Independent Effects of ER-α36 in ER-Negative Breast Cancer, Steroids. (2012) 77, no. 6, 666–673, 10.1016/j.steroids.2012.02.013. 22402113

[32] Wishart A. L. , Conner S. J. , Guarin J. R. et al., Decellularized Extracellular Matrix Scaffolds Identify Full-Length Collagen VI as a Driver of Breast Cancer Cell Invasion in Obesity and Metastasis, Science Advances. (2020) 6, no. 43, 10.1126/sciadv.abc3175. PMC7577726 33087348

[33] Solsona-Vilarrasa E. and Vousden K. H. , Obesity, White Adipose Tissue and Cancer, FEBS Journal. (2025) 292, no. 9, 2189–2207, 10.1111/febs.17312. 39496581 PMC12062788

[34] Destounis S. , Newell M. , and Pinsky R. , Breast Imaging and Intervention in the Overweight and Obese Patient, American Journal of Roentgenology. (2011) 196, no. 2, 296–302, 10.2214/ajr.10.5556. 21257879

[35] Mann R. M. , The Effectiveness of MR Imaging in the Assessment of Invasive Lobular Carcinoma of the Breast, Magnetic Resonance Imaging Clinics of North America. (2010) 18, no. 2, 259–276, 10.1016/j.mric.2010.02.005. 20494311

[36] Albayrak Z. K. , Onay H. K. , Karatağ G. Y. , and Karatağ O. , Invasive Lobular Carcinoma of the Breast: Mammographic and Sonographic Evaluation, Diagnostic and interventional radiology. (2011) 17, no. 3, 232–238, 10.4261/1305-3825.Dir.598-06.3. 20706979

[37] Sezgin G. , Apaydin M. , Etit D. , and Atahan M. K. , Tumor Size Estimation of the Breast Cancer Molecular Subtypes Using Imaging Techniques, Med Pharm Rep. (2020) 93, no. 3, 253–259, 10.15386/mpr-1476. 32832890 PMC7418834

[38] Browne R. , McAnena P. , O’Halloran N. et al., Preoperative Breast Magnetic Resonance Imaging as a Predictor of Response to Neoadjuvant Chemotherapy, Breast Cancer: Basic and Clinical Research. (2022) 16, 1–11, 10.1177/11782234221103504. PMC9234834 35769423

[39] O′Connell A. , Conover D. L. , Zhang Y. et al., Cone-Beam CT for Breast Imaging: Radiation Dose, Breast Coverage, and Image Quality, American Journal of Roentgenology. (2010) 195, no. 2, 496–509, 10.2214/ajr.08.1017. 20651210

[40] Haloua M. H. , Krekel N. M. , Coupé V. M. et al., Ultrasound-Guided Surgery for Palpable Breast Cancer Is Cost-Saving: Results of a Cost-Benefit Analysis, Breast. (2013) 22, no. 3, 238–243, 10.1016/j.breast.2013.02.002. 23478199

[41] Duan Y. , Guo D. , Zhang X. et al., Diagnostic Accuracy of Optical Coherence Tomography for Margin Assessment in Breast-Conserving Surgery: A Systematic Review and Meta-Analysis, Photodiagnosis and Photodynamic Therapy. (2023) 43, 10.1016/j.pdpdt.2023.103718. 37482370

[42] Wang J. , Zhang L. , and Pan Z. , Evaluating the Impact of Radiofrequency Spectroscopy on Reducing Reoperations After Breast Conserving Surgery: A Meta-Analysis, Thoracic Cancer. (2023) 14, no. 16, 1413–1419, 10.1111/1759-7714.14890. 37073138 PMC10234782

[43] Boughey J. C. , Hieken T. J. , Jakub J. W. et al., Impact of Analysis of Frozen-Section Margin on Reoperation Rates in Women Undergoing Lumpectomy for Breast Cancer: Evaluation of the National Surgical Quality Improvement Program Data, Surgery. (2014) 156, no. 1, 190–197, 10.1016/j.surg.2014.03.025. 24929768

[44] Jorns J. M. , Visscher D. , Sabel M. et al., Intraoperative Frozen Section Analysis of Margins in Breast Conserving Surgery Significantly Decreases Reoperative Rates: One-Year Experience at an Ambulatory Surgical Center, American Journal of Clinical Pathology. (2012) 138, no. 5, 657–669, 10.1309/ajcp4iemxcj1gdts. 23086766 PMC3988579

